# Neutrophil Extracellular Traps and Thrombolysis Resistance: New Insights for Targeting Therapies

**DOI:** 10.1161/STROKEAHA.123.045225

**Published:** 2024-03-11

**Authors:** Luca Mengozzi, Ilaria Barison, Martin Malý, Giulia Lorenzoni, Marny Fedrigo, Chiara Castellani, Dario Gregori, Petr Malý, Radoslav Matěj, Petr Toušek, Petr Widimský, Annalisa Angelini

**Affiliations:** Cardiac Centre (L.M., P.T., P.W.), Charles University, Czechia.; Department of Pathology (R.M.), Charles University, Czechia.; Third Faculty of Medicine and University Hospital Královské Vinohrady, Military University Hospital in Prague, First Faculty of Medicine (M.M., P.M.), Charles University, Czechia.; Cardiovascular Pathology (I.B., M.F., C.C., A.A.), Department of Pathology, Cardiac, Thoracic and Vascular Sciences, Public Health, University of Padua, Italy.; Unit of Biostatistics, Epidemiology, and Public Health (G.L., D.G.), Department of Pathology, Cardiac, Thoracic and Vascular Sciences, Public Health, University of Padua, Italy.

**Keywords:** extracellular traps, immunohistochemistry, ischemic stroke, myocardial infarction, pathology

## Abstract

**BACKGROUND:**

Thrombosis is linked to neutrophil release of neutrophil extracellular traps (NETs). NETs are proposed as a mechanism of resistance to thrombolysis. This study intends to analyze the composition of thrombi retrieved after mechanical thrombectomy, estimate the age and organization of thrombi, and evaluate associations with the use of thrombolysis, antiplatelets, and heparin.

**METHODS:**

This retrospective observational study involved 72 samples (44 from cerebral and 28 coronary arteries), which were stained with hematoxylin and eosin, anti-NE (neutrophil elastase) antibody, and anti-histone H2B (histone H2B) antibody, representing different components in NET formation, all detectable during the later stages of NETosis, for histochemical and digital quantification of NET content. The histological and morphological evaluations of the specimens were correlated, through univariate and mediation analyses, with clinical information and therapy administered before intervention.

**RESULTS:**

The results demonstrated that the composition of cerebral and coronary thrombi differs, and there were significantly more lytic cerebral thrombi than coronary thrombi (66% versus 14%; *P*=0.005). There was a considerably higher expression of NETs in the cerebral thrombi as testified by the higher expression of H2B (*P*=0.031). Thrombolysis was remarkably associated with higher NE positivity (average marginal effect, 6.461 [95% CI, 0.7901–12.13]; *P*=0.02555), regardless of the origin of thrombi. There was no notable association between the administration of antiaggregant therapy/heparin and H2B/NE amount when adjusted for the thrombus location. Importantly, the age of the thrombus was the only independent predictor of NET content without any mediation of the thrombolytic treatment (*P*=0.014).

**CONCLUSIONS:**

The age of the thrombus is the driving force for NET content, which correlates with impaired clinical outcomes. The therapy that is currently administered does not modify NET content. This study supports the need to investigate new pharmacological approaches added to thrombolysis to prevent NET formation or enhance their disruption, such as recombinant human DNase I (deoxyribonuclease I).

Strokes remain one of the leading causes of death and disability worldwide.^1^ Intravenous thrombolytic therapy has proved to be effective only in a relatively small percentage of patients with stroke and recombinant tissue-type plasminogen activators (r-tPAs) may become an obsolete and ineffective treatment if results are not improved.^2^ While there is still considerable debate on the use of thrombolytics among clinicians, one clear explanation of their inefficacy in coronary ischemia and cerebral infarctions lies in the composition of the thrombi, which shows resistance to medical therapy due to the nature of their cellular and extracellular components. According to histological studies, cardioembolic thrombi showed a higher amount of neutrophils, a higher proportion of fibrin/platelets, and fewer erythrocytes than noncardioembolic thrombi.^3^

Thrombosis has recently been linked to neutrophil activation and release of neutrophil extracellular traps (NETs) via a process called NETosis.^4^

Neutrophils promote thrombus formation by 2 different mechanisms, from one side by localizing procoagulant factors including tissue factor and factor XII and on the other side by the formation of NETs, which are created when activated neutrophils release decondensed DNA fragments into the extracellular space.^5^ This process is called NETosis. NETs are networks of extracellular fibers, primarily composed of DNA from neutrophils.^6^ NETs are composed of a processed type of chromatin, tethered to specific cytoplasmic proteins with granular features.^7^ In naive neutrophils, granular and nuclear antigens are separated, and within the process of NETosis, some granular proteins, such as myeloperoxidase and NE (neutrophil elastase), gradually migrate to the nucleus.^8^ In addition, histones are citrullinated during NETosis.^9,10^ The position of nuclear granular proteins and the citrullination of histones offer unique characteristics for neutrophils undergoing NETosis that can be used for their identification in specimens.^8^ NETs can be identified by the presence of extracellular DNA–histone complexes (H2B) and NE.

The age of thrombi was extensively studied in acute myocardial infarctions (MIs); however, less is known about the age of thrombi in acute ischemic strokes (ISs). In the literature,^5,10^ the presence of NETs varies according to the age of the thrombus, with fewer NETS in the fresh thrombus during its formation, increasing progressively in the lytic phase and disappearing in the organized thrombus, relating the age of the thrombus with the presence and number of NETs. Therefore, a clearer understanding of the time of clots formation, also according to the number of NETs within the retrieved specimen, could set the basis for risk assessments and better therapeutic treatment in clinical scenarios.

In this study, we analyzed in depth the composition of thrombi retrieved after mechanical thrombectomy (MT) in patients treated at 2 stroke units in Prague. By means of pathological characterization of NETs, ensuring their proper detection and confirming their origin from leucocytes, we selected 2 antibodies targeting NE and H2B and representing different components in NET formation, both detectable during the later stages of NETosis. We quantified the number of NETs, and we compared them to thrombi retrieved during coronary artery thrombus aspiration in MI. We also assessed the age and organization of thrombi and quantified NETs in relation to the use of anticoagulants (heparin), antiplatelet, and thrombolytics.

## METHODS

The data that support the findings of this study are available from the corresponding author upon reasonable request.

### Study Population Selection, Therapy, Sample Collection, and Processing

Thrombi were retrieved over 6 months from patients undergoing MT or percutaneous coronary intervention (PCI) at the Cardiac Center of the University Hospital Královské Vinohrady in Prague and the Cerebrovascular Center of the Military University Hospital in Prague. All specimens were derived from patients providing informed consent to the analysis of biological material retrieved during the required intervention, and the study had been formally approved by the ethics committee of the University Hospital Královské Vinohrady. No exclusion criterion was adopted in the selection of patients for the purpose of comparing as many specimens as possible and to obtain a real-world sample of a 6-month time frame medical practice. Patients requiring MT for acute IS or PCI for MI received standard care, and no alternative protocol was used. Specimens were retrieved only if standard care allowed it. Only patients without contraindications to the use of thrombolysis were treated with r-tPA. Anticoagulant and antiaggregant therapies were administered only if necessary and to patients without contraindications. All patients with acute MI received the acute phase 3 antithrombotic drugs: intravenous lysin salicylate, 500 mg; unfractionated heparin, 100 units/kg; and ticagrelor, 180 mg. All patients with stroke receiving thrombolytic treatment did not receive any other antithrombotic drug during the acute phase before thrombus removal. Patients with stroke, undergoing direct MT (without bridging thrombolysis), received a minimal dosage of unfractionated heparin (10–25 units/kg) during the procedure. Specimens retrieved after MT or PCI were immediately preserved in a 10% formalin solution at 4 °C and carried within 2 hours to the laboratory for paraffin embedding.

### Histology and Estimation of Thrombus Age

Thrombi specimens were formalin-fixed, paraffin-embedded, and cut in 5 µm with an LEICA SM2010R microtome. Samples were fixed by using the tissue processor VIP 6 (SAKURA). Tissues were deparaffinated, rehydrated, and stained with hematoxylin and eosin to assess the morphology and the age of the sample. Two expert pathologists conducted a detailed blinded morphological semiquantitative analysis, by assessing the presence of leucocytes, Zahn lines, fibrosis, atheromatous material, and fibrin-platelet complexes^.11,12^ The semiquantification of each component was expressed as a percentage of the single component on the total area of the thrombus.

Specimens were classified based on their age, according to the criteria reported by Rittersma et al,^13^ which represent the criteria followed afterward in all the papers dealing with thrombus material retrieved by coronary and cerebral arteries. We, too, have endorsed their classification, which we are reporting as follows. Fresh thrombi (<1 day) are characterized by layered patterns of platelets, fibrin, erythrocytes, and intact granulocytes. Lytic thrombi (1–5 days) present an area of colliquative necrosis and Karyorrhexis of granulocytes. Organized thrombi (>5 days) show ingrowth of smooth muscle cells, with or without depositions of connective tissue and capillary vessel ingrowth. As suggested by these authors, samples that have a heterogeneous composition are scored according to the age of the older part.

### Immunohistochemistry and NET Quantification

Two tissue sections for each sample were immunoassayed to identify and quantify NETs through anti-NE and anti-H2B antibodies. They were selected because they represent different components in NET development, ensuring proper detection of NETs and confirming their origin from leucocytes. NE is a serine protease that during NETosis translocates into the nucleus and cleaves histones, such as H2B, and facilitates the homogenization of euchromatin and heterochromatin.^14^

Sections were cut on a LEICA SM2010R slide microtome and were deparaffinized and rehydrated, and 1 section for each sample was incubated in anti-NE (Millipore 481001: Rabbit pAb, 1 mL). The immunohistochemical staining was performed with the automated Ventana BenchMark ULTRA from ROCHE, and all reagents used, except the primary antibody, are from ROCHE. The primary antibody was diluted by 1:50 in an antibody diluent from DAKO (Dako-Agilent S0809 ready-to-use). Antigen retrieval was carried out at 37 °C with a citrate buffer at pH 6. The secondary antibody is from the ROCHE OptiView DAB Detection Kit (protocol U Optiview DAB IHC, v6). The second section was stained with an Anti-Histone H2B antibody (ab134211: Abcam, 100 µg). The primary antibody was diluted by 1:500 in an antibody diluent from DAKO (Dako-Agilent S0809 ready-to-use). The secondary antibody (Goat-Chicken IgY ab97135) was diluted by 1:2000 (Dako-Agilent S0809 ready-to-use). Heat-mediated (60 °C) antigen retrieval was performed with a citrate buffer solution at pH 6. Stained slides were scanned with Aperio ScanScope CS and analyzed with QuPath software,^15^ v0.3.0, to quantify NE and H2B content, expressed as the percentage of the total area that stains positive for the NE or H2B.

### Statistical Analysis

Descriptive statistics were reported as median (I quartile; III quartile) for continuous variables and as absolute numbers as corresponding percentages for categorical variables. Wilcoxon and χ^2^ tests were performed to compare the distribution of continuous and categorical variables, respectively. *P* values underwent Benjamini-Hochberg correction to account for the multiplicity of testing. The association between the type of therapy administered and the NET content was evaluated using gamma regression because NETs were found to be not normally distributed according to the Shapiro-Wilk test. The marginal effect was computed considering the partial derivatives of the marginal expectation. Results were reported as an average marginal effect, 95% CI, and *P* value. Mediation analysis^16^ was performed to disentangle the effect of thrombosis site and age on NET content. CIs were computed using a nonparametric bootstrap with 5000 draws. Analyses were performed using the R software^17^ within the packages mediation,^18^ regression modeling strategies, and margins.

## RESULTS

### Population Risk Factors and Pharmacological Therapy in Cerebral Versus Coronary Thrombi

The study included 72 thrombotic/embolic specimens: 28 retrieved from coronary and 44 from cerebral arteries. We evaluated the clinical data in both groups based on the thrombosis site (cerebral versus coronary). This analysis showed differences in cardiovascular risk factors prevalence (body mass index, patients’ age, smoking, hypertension, and previously reported coronary disease) but no major differences in terms of chronic therapy before acute events (Table 1). Conversely, the therapy administered during thrombectomy differs, according to guidelines, with a higher prevalence of heparin and antiaggregant in coronary thrombosis and a definite more frequent administration of thrombolysis in patients with cerebral stroke. Vascular risk factors are different between coronary and cerebral thrombi. This reflects the differences in the cause and pathophysiology of MI and IS. While MI is typically caused by atherothrombosis (ie, in situ thrombus on an unstable plaque), large vessel occlusion IS is typically caused by cardioembolism (eg, in atrial fibrillation) and, thus, unrelated to atherosclerosis. This difference may explain why most cerebral thrombi are older and less fresh than coronary thrombi.

**Table 1.**
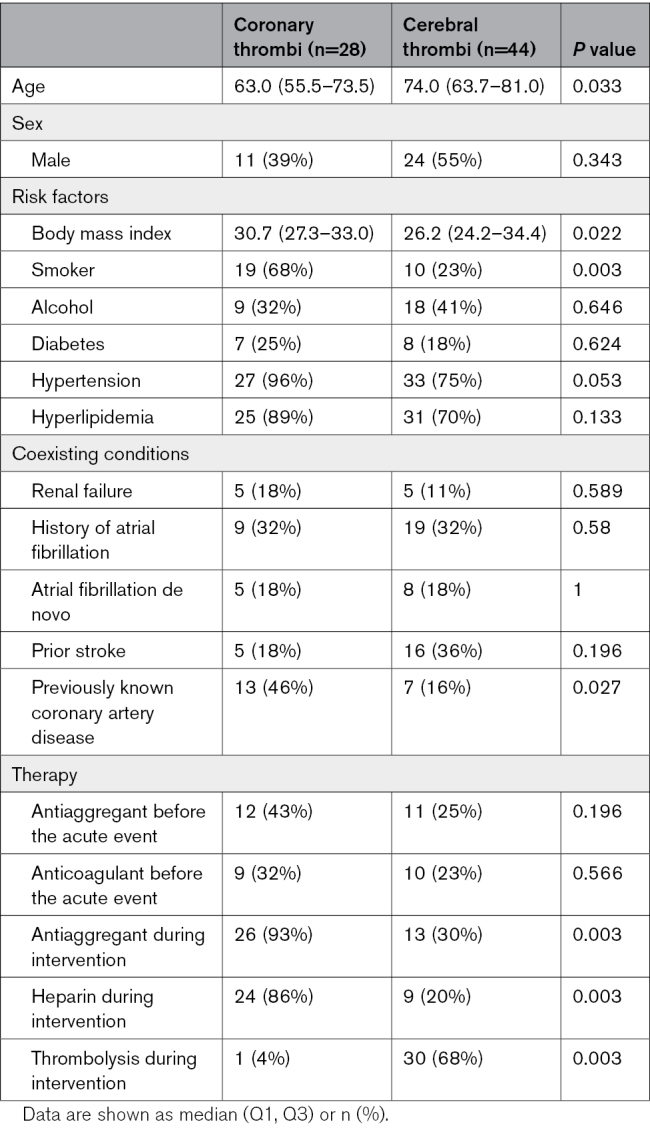
Patients’ Clinical Characteristics of Coronary Versus Cerebral Thrombi

### Histological Classification, Age Assessment, and NET Quantification

**Figure 1.**
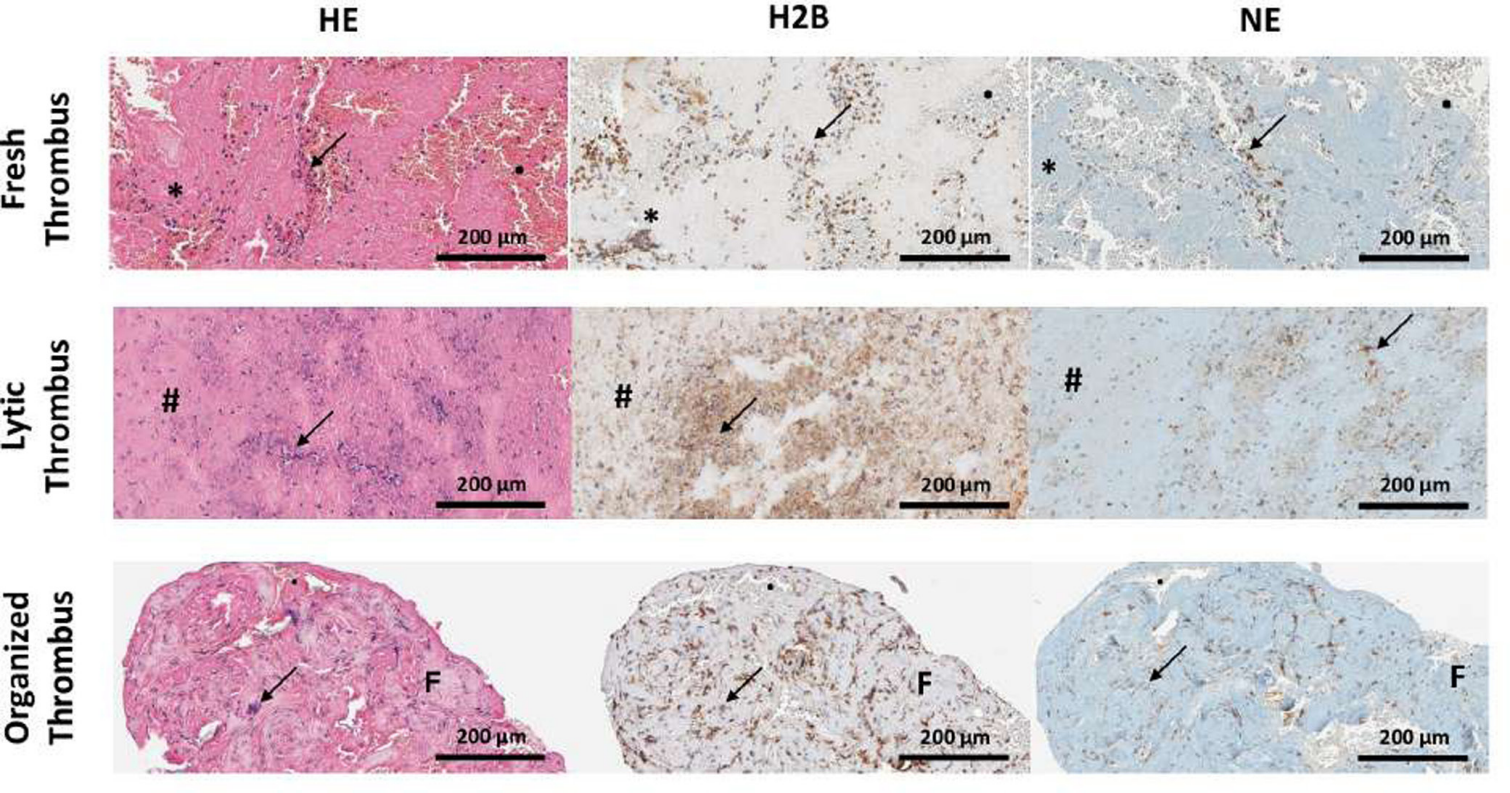
**Three differently aged thrombi. A**, Fresh thrombus (aged <1 d). **B**, Lytic thrombus (aged 1–5 d). **C**, Organized thrombus (aged >5 d). Each specimen was stained with hematoxylin and eosin (HE) to identify each component and immunoassayed with anti-H2B and anti-NE (neutrophil elastase) antibodies for detecting neutrophil extracellular traps (NETs). Note in (**A**) Layered patterns of platelets, fibrin, intact erythrocytes, and granulocytes, and a low NET presence. Note in (**B**) colliquative necrosis and Karyorrhexis of granulocytes with a higher number of NETs. Note in (**C**) an organized thrombus (>5 d) showing ingrowth of fibroblasts and capillary vessels. Asterisks indicate Zahn lines, arrows indicate leucocytes, black dots indicate erythrocytes, hashtags indicate fibrin-platelet complexes, and F indicates fibrosis. Scale bar: 200 µm.

Specimens were histologically evaluated to classify their age, according to Rittersma et al,^13^ and 54% of them were fresh (84% of the coronary and 34% of the cerebral thrombi), while 46% were lytic (14% of the coronary and 66% of the cerebral thrombi; *P*=0.005). There were only 4 organized thrombi that were discarded from the analysis. In fact, we wanted to guarantee the robustness and reliability of the results, as their limited sample size could potentially introduce undue bias in our findings. Focusing on the typical morphological and cellular component of the samples, the analysis showed a higher presence of leucocytes (*P*<0.001), fibrin platelets (*P*=0.032), and Zahn lines (*P*=0.027) in cerebral thrombi compared with coronary specimens. Moreover, the cerebral samples revealed an increase in H2B positivity (*P*=0.031), lower erythrocyte aggregates (*P*=0.032), and the absence of atheromatous material (*P*=0.01; Table 2; Figure 1).

**Table 2.**
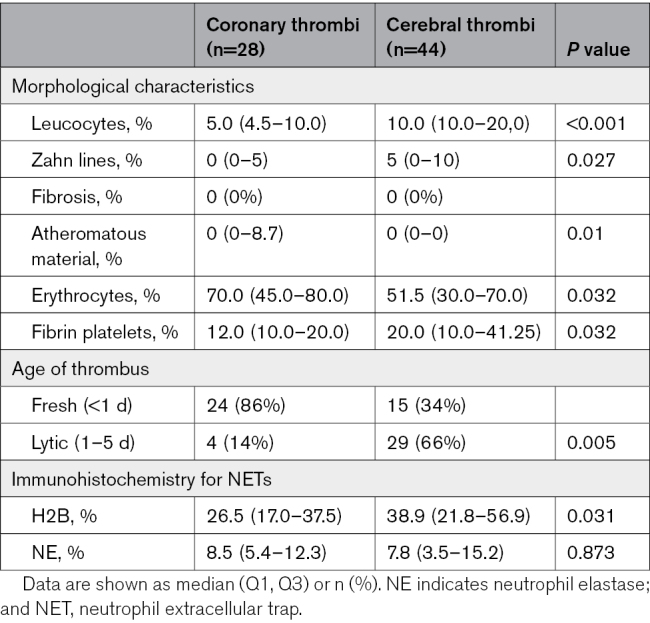
Histomorphological Characteristics of Coronary Versus Cerebral Thrombi

### Association of Therapy With NET Content

The main aim of this study was to assess the association of the pharmacological therapy administered before and during procedural interventions with NET content. The univariable analysis of the impact of the drugs administered during the interventional procedures on the number of NETs is shown in Table 3. The results showed a statistically significant association between thrombolysis and a higher level of NE positivity (average marginal effect, 6.461 [95% CI, 0.7901–12.13]; *P*=0.02555). In addition, the administration of an antiaggregant is associated with a lower level of H2B (*P*=0.0311) and NE positivity (*P*=0.035), whereas the use of heparin is associated with a lower level of H2B (*P*=0.03574). Because the pharmacological approach is significantly different in cerebral and coronary thrombi, we adjusted the univariate analyses for the thrombosis site (cerebral versus coronary thrombi) to account for potential confounding factors. All the associations were no more significant after the adjustment for the thrombosis site, except for thrombolysis and NETs (average marginal effect, 7.896 [95% CI, 0.2095–15.58]; *P*=0.04407).

**Table 3.**
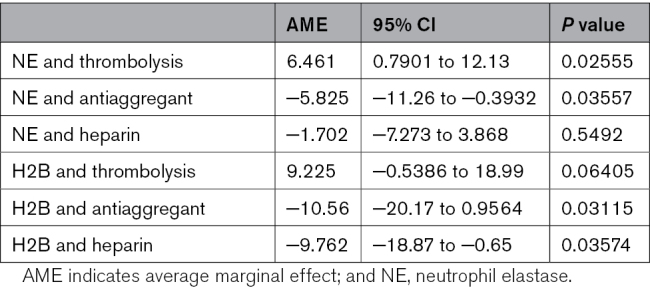
Univariable Analysis of the Association Between NE and Therapy and Univariable Analysis of the Association Between H2B and Therapy

### Association Among Age, Thrombolysis, and NET Content

**Figure 2.**
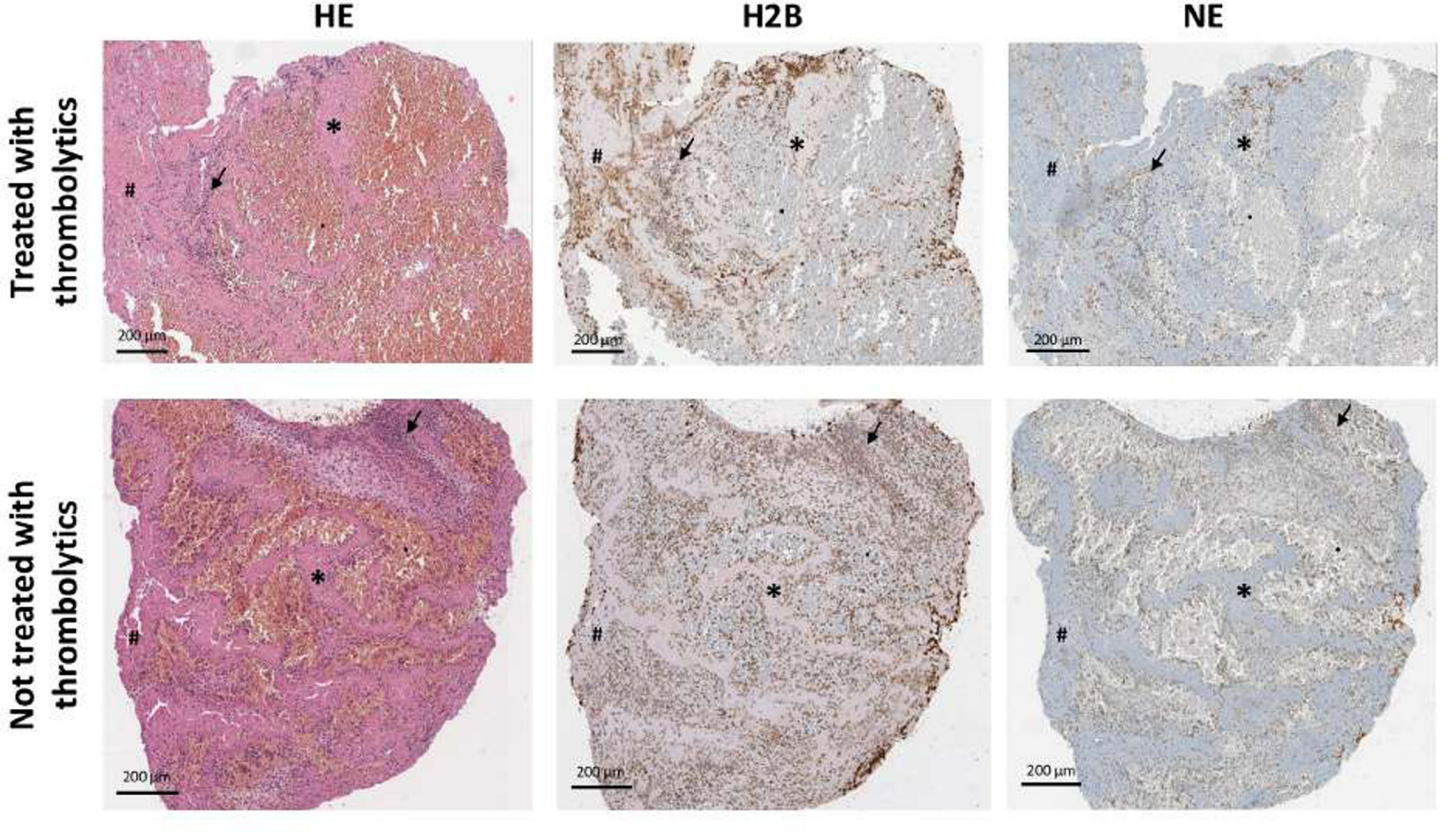
**Morphology of a thrombus treated and a thrombus not treated with thrombolytics.** Each specimen is stained with hematoxylin and eosin (HE) and immunoassayed with anti-H2B and anti-NE (neutrophil elastase) antibodies for detecting neutrophil extracellular traps (NETs). Asterisks indicate Zahn lines, arrows indicate leucocytes, black dots indicate erythrocytes, and hashtags indicate fibrin-platelet complexes. The comparison among these 2 groups does not reveal a significantly different morphology. Scale bar: 200 µm.

**Figure 3.**
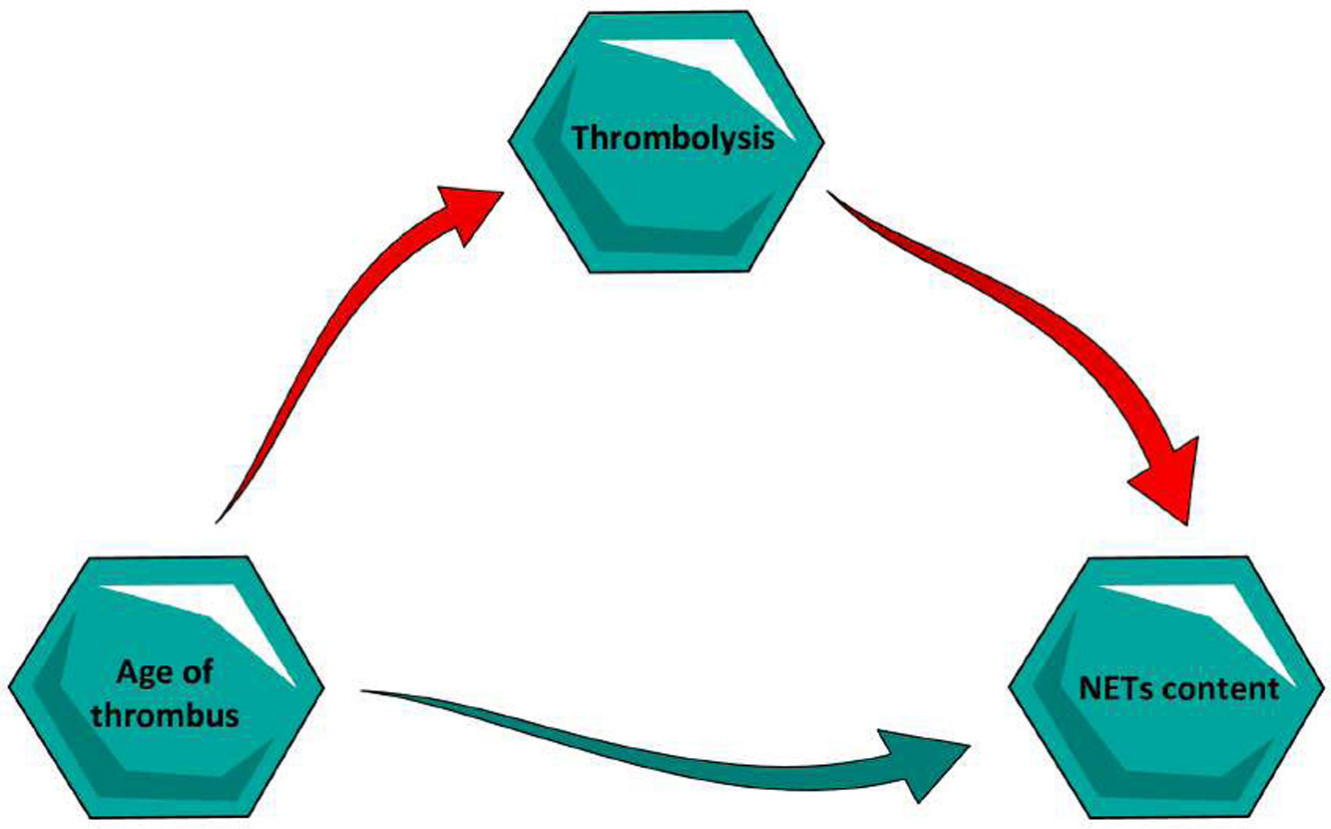
**Schematic representation of mediation analysis hypothesis.** Thrombolysis does not have any mediation effect on the association between the age of the thrombus and neutrophil extracellular trap (NET) content.

Thrombolysis, as a variable, resulted to be significantly associated with a higher content of NETs even after adjustment for the thrombosis site; thus, we deeply investigated the clinical and morphological characteristics of our population, comparing the thrombolysis-treated group (n=31) with the patients not treated with thrombolysis (n=41; Table S1; Figure 2). This analysis of clinical and morphological characteristics did not show significant differences, except for the age of the thrombus, which is the only variable acting on the NET content (Tables S1 and S2). Thus, there was a higher prevalence of lytic thrombi (>1 day) in the thrombolytic group (*P*=0.03). These results suggested that the ages of the thrombus and r-tPA were both associated with the NET content. For this reason, we performed a further evaluation of the association between thrombolysis and NE positivity adjusting for the age of the thrombus. The age of the thrombus proved to be the only independent predictor of the NE, while thrombolysis lost its significance on the NET contents. Furthermore, we tested whether thrombolysis had a mediation effect on the association between the age of the thrombus and the content of NETs (Figure 3). The mediation analysis revealed that the age of the thrombus had a direct effect on the NET content (*P*=0.014), without any mediation of the thrombolysis.

## DISCUSSION

The main results of this study demonstrated that the composition of cerebral and coronary thrombi differs, and there were significantly more lytic cerebral thrombi than coronary thrombi. Moreover, there were more leukocytes, fibrin/platelets, and Zahn lines and fewer erythrocytes in cerebral thrombi compared with coronary thrombi. No atheromatous material was found in cerebral thrombi, confirming the main embolic origin of cerebral thrombi. Interestingly, there was a considerably higher expression of NETs in the cerebral thrombi as testified by the higher expression of H2B. Thrombolysis was associated with higher NE positivity, regardless of the origin of the thrombi (ie, cerebral versus coronary thrombi). There was no notable association between the administration of antiaggregant/heparin and H2B/NE amount when adjusted for the thrombus location. Importantly, the age of the thrombus was the only independent predictor of NET content without any mediation of the thrombolytic treatment.

Our samples were fixed, and the NET components were marked by immunohistochemistry staining with anti-H2B to highlight the histone elements and anti-NE to determine the neutrophil origin of these extracellular structures. Our study includes samples retrieved from different thrombotic sites, allowing us to evaluate the possible effects of the thrombotic site itself on morphology and NET content, as suggested in the literature.^19^

First, we directed our attention to the evaluation of the age of retrieved thrombi, and we observed that, in our population, the cerebral specimens presented the largest level of thrombus maturation and a higher presence of leucocytes, in agreement with previous studies on IS thrombi with less NETs in fresh thrombi and a progressive increase during the lytic phase. Indeed, Laridan et al^5^ quantified neutrophils in stroke thrombi, demonstrating that their amount is considerably higher when compared with coronary or stent thrombi.^20^ We assessed the differential effect of the various therapies on the composition of the specimens. The difference in the pharmacological treatment between the 2 groups led us to perform an adjusted analysis to remove confounding factors and strengthen the results.

Many studies demonstrated that NETs have a relevant role in thrombosis, with a procoagulant effect,^21,22^ and their presence in retrieved thrombotic/embolic material is associated with impaired clinical outcome.^19^ Thrombolysis sensitivity is associated with fibrin-clot architecture in the thrombus. Fresh thrombi are typically associated with improved outcomes such as higher Thrombolysis in Cerebral Infarction scores, shorter procedural time, and increased sensitivity to thrombolysis. Conversely, fibrin-rich thrombi, because of their higher stiffness, show increased resistance to MT and thrombolysis. Recent studies^23–25^ claim that the outer layer of thrombi retrieved from patients with stroke is composed mainly of RBC, fibrin, von Willebrand Factor, and leukocytes that all play a role against r-tPA. An improvement in the rate of r-tPA effectivity is directly proportional to this external shell being compromised. Thus, targeting components other than fibrin can cause breaches into the outer layer of thrombi, which can enhance the efficacy of thrombolytics. An important consideration for clinical practice is that for every 5.4 patients treated with MT, 1 less patient will be dependent at 90 days, whereas for thrombolysis, the number needed to treat is 18, that is, 18 patients with stroke have to be administered thrombolysis to obtain a clinical benefit for 1 patient. In brief, MT is 3.3× more effective than thrombolysis.^26^ The risk of complications related to stroke thrombectomy is higher in small distal arteries (eg, M2 segment), and the procedural success rate is smaller in these distal segments; thus, the difference against thrombolysis is minimal. While for large vessel occlusion stroke (typically M1 segment of medial cerebral artery), the number needed to treat by thrombectomy to achieve functional independence is around 5,^27^ for small vessels (eg, M2), it is not known; for primary PCI in STEMI to prevent death, the number needed to treat is 29.^28^ Although unfractionated heparin, along with other intravenous anticoagulants, holds a key role in the treatment of acute coronary syndromes, it is contraindicated, for the most part, in acute IS. There are several reasons for contraindication of full therapeutic anticoagulants in stroke: hemorrhagic stroke (15% of all strokes, frequently difficult to differentiate based only on clinical signs), risk of hemorrhagic transformation of IS (especially large IS, ie, those suitable for thrombectomy are at risk), uncontrolled hypertension, preexisting anticoagulant therapy, etc.^29^ Reperfusion damage is closely related to sudden vigorous blood flow restoration to an ischemically damaged tissue; thus, it could be considered as reperfusion hemorrhage. This is rarely clinically relevant in acute MI, where the connective tissue network is robust enough; thus, in the myocardium, large reperfusion benefits almost always overcome small reperfusion damage. The situation in the brain is different, mainly due to far less robust connective tissue; thus, reperfusion damage is a serious concern for the brain. This difference is reflected in the heparin use during the acute phase of MI (dosage of 70–100 units/kg is standard) as opposed to acute IS, where heparin is considered a class III indication, that is, it should not be used and certainly not in full therapeutic dose. When heparin is used in acute IS mainly to prevent catheter clots, the dose should be only around 20 to 30 units/kg. The difference in thrombolysis use in MI versus IS involves a complex explanation. In brief, in MI, thrombolysis does not add any benefit on top of mechanical reperfusion, and the so-called facilitated PCI was demonstrated to be inferior to simple direct PCI. This may be related to procedural techniques (high-pressure balloon dilatation and permanent stent implantation). Trials comparing direct MT versus bridging thrombolysis before MT demonstrated similar outcomes or even better outcomes with bridging thrombolysis. The differences in the use of thrombolysis may explain (at least partially) the clot features in Table 2 and Table S1.

Many studies reported that heparin has an inhibitory effect on the interaction between NET histone components and platelets.^22,30,31^ Accordingly, we detected a higher presence of NETs, marked with H2B, in the thrombolysis-treated group. Because these results suggested that the ages of the thrombus and r-tPA were both associated with a higher number of NETs, we tested the impact of thrombolysis on the association between the age of the thrombus and the NET specimen content. We demonstrated that the age of the thrombus has a direct effect on NET content, without any mediation of thrombolysis.

The main limitations of the study are related to the small sample size and the absence of organized thrombi, older than 5 days. Unfortunately, the cases are not representative of older thrombi morphological features; however, our main interest was evaluating the morphology and the therapeutical effects in the acute and subacute phases. Samples are equally distributed in the 2 classes of fresh (<1 day) and lytic (1–5 days) thrombi. For the first time, these mediation analysis results indicate a causal effect of the age of the thrombus on the NET bulk. The small availability of organized thrombi is caused mainly by the fact that patients entering the study were only those who, in a specific time frame, experienced an IS or MI and required an MT or PCI. Specimens were retrieved over a period of 6 months and represent the real-world sample of a specific time frame alongside probability factors. The city of Prague offers a high density of stroke units, and tertiary and quaternary care facilities, and the time needed to treat a symptomatic patient is considerably reduced.^24^ Another possible limitation of such a study is the content of NETs retrieved after MT, thus, analyzed and the amount that could have been left within the vasculature of the patients; nevertheless, for patients with IS, each individual in this study scored between 2b and 3 on the Thrombolysis in Cerebral Infarction scale, when evaluated after MT, determining, then, an angiographic success in the whole group.^25,32^

These results provide evidence of the requirement to improve the current pharmacological protocols during acute stroke. Although it is known that heparin has a partial inhibitory effect on NET maturation, the current therapy formulation is not sufficient to contrast their procoagulant effect and resistance to thrombolysis. Adding new drugs with a specific action to disaggregate chromatin and enzymatic components of NETs can enhance the lytic effects of the therapy and improve patients’ outcomes. Presently, DNase I (deoxyribonuclease I) has shown remarkable results in targeting NETS and extracellular DNA ex vivo lysis, thus demonstrating prothrombolytic potential when coupled with current thrombolytics.^5,33,34^ Recombinant human DNase I, under the name of dornase alfa, is currently labeled as a treatment in cystic fibrosis; dornase alfa hydrolyzes DNA present in the secretions of the airways in patients with cystic fibrosis, reducing viscosity, improving quality of life, and reducing mortality by infection.^33,35^

In conclusion, this study provides valuable insights into the complex relationship between thrombus composition, age, pharmacological therapy, and NET content and demonstrates that the age of the thrombus is the only independent predictor of NET content without any mediation of the thrombolytic treatment in thrombotic/embolic specimens retrieved during thrombectomy in patients with stroke. This study supports the need for further investigations in also pharmacologically targeting NETs found in patients with acute cerebral ischemia, possibly, with recombinant human DNase I.

## ARTICLE INFORMATION

### Acknowledgments

The authors thank the medical staff of the Cardiac Center, University Hospital Královské Vinohrady, Prague, Czechia, specifically the interventionalists and the nurses from interventional cardiology and angiology for providing specimens after mechanical thrombectomy (MT) and percutaneous coronary intervention (PCI). The authors also thank the Cerebrovascular Center, Military University Hospital Prague, specifically the interventionalists and the nurses for also retrieving and providing specimens after MT and PCI. Furthermore, the authors thank the staff of the Department of Pathology, University Hospital Královské Vinohrady, and the Cardiovascular Pathology, Department of Cardiac, Thoracic, Vascular Sciences and Public Health, University of Padua, Padua, Italy, for their qualified laboratory assistance with the handling of the specimens. The graphical abstract was generated using Servier Medical Art, provided by Servier, licensed under a Creative Commons Attribution 3.0 Unported license.

### Sources of Funding

This work was supported by the Cardiovascular Research Program Cooperatio, Charles University, Prague, Czechia; the Third Faculty of Medicine, Charles University; and the Grantova Agentura Univerzity Karlovy (Charles University Grant Agency) Project No. 940120, Department of Science and Research, Czechia.

### Disclosures

None.

### Supplemental Material

STROBE Checklist

Tables S1–S2

## Supplementary Material


