# Supplementary material for: Neutrophil Extracellular Traps and Thrombolysis Resistance: New Insights for Targeting Therapies
Source: Stroke. 2024 Mar 11;55(4):963–71. doi: 10.1161/STROKEAHA.123.045225 (PMC10962437; doi:10.1161/STROKEAHA.123.045225)
Supplement: Supplementary file 1 [file str-55-0963-s001.pdf]

Table S1. Patients' clinical characteristic of not treated with thrombolysis group vs thrombolysis treated group. Data are shown as median [Q1;Q3] or n (%).

	Not treated with thrombolysis (n=41)	Treated with thrombolysis (n=31)	p-value
Age	64.0 [57.0;75.0]	73.0 [63.5;81.5]	0.207
Sex			
Male	20 (49%)	15 (48%)	1
Risk factors			
BMI	29.3 [25.5;32.9]	26.3 [24.2;34.4]	0.215
Smoker	22 (54%)	7 (23%)	0.092
Alcohol	13 (32%)	14 (45%)	0.653
Diabetes	10 (24%)	5 (16%)	0.907
Hypertension	37 (97%)	23 (74%)	0.316
Hyperlipidemia	35 (85%)	21 (68%)	0.267
Coexisting conditions			
Renal failure	35 (85%)	27 (87%)	1
History of atrial fibrillation	15 (37%)	13 (42%)	1
Atrial fibrillation *de novo*	6 (15%)	7 (23%)	0.907
Stroke	9 (22%)	12 (39%)	0.498
Coronary artery disease	17 (41%)	3 (10%)	0.029
Therapy			
Antiaggregant prior to the acute event	14 (34%)	9 (29%)	1
Anticoagulant prior to the acute event	14 (34%)	5 (16%)	0.316
Antiaggregant during PCI	37 (90%)	2 (6%)	0.007
Heparin during PCI	29 (71%)	4 (13%)	0.007

Table S2. Histomorphological characteristics of not treated with thrombolysis group vs thrombolysis treated group. Data are shown as median [Q1;Q3] or n (%).

	Not treated with thrombolysis (n=41)	Thrombolysis treated (n=31)	p-value
Morphological characteristics			
Leucocytes (%)	10 [5;15]	10 [7;15]	0.266
Zahn lines (%)	5 [0;5]	5 [0;0]	0.217
Fibrosis (%)	0 (0%)	0 (0%)	
Atheromatous material (%)	0 [0;0]	0 [0;0]	0.394
Erythrocytes (%)	65 [40;78]	60 [30;70]	0.394
Fibrin-platelets (%)	15.0 [10.0;25.0]	20.0 [11.5;42.5]	0.217
Age of thrombus			
Fresh (<1 day)	28 (68%)	11 (35%)	
Lytic (<1 day; >5days)	13 (32%)	20 (65%)	0.03
Immunohistochemistry for NETs			
H2B (%)	26.1 [17.5;41.4]	38.2 [25.2;54.3]	0.06405
NE (%)	7.94 [4.3;11.1]	8.16 [3.4;24.9]	0.02555
